# Prognostic Value of an Inflammation-Related Index in 6,865 Chinese Patients With Postoperative Digestive Tract Cancers: The FIESTA Study

**DOI:** 10.3389/fonc.2019.00427

**Published:** 2019-05-22

**Authors:** Xinran Zhang, Dan Hu, Xiandong Lin, Hejun Zhang, Yan Xia, Jinxiu Lin, Xiongwei Zheng, Feng Peng, Jianzheng Jie, Wenquan Niu

**Affiliations:** ^1^Institute of Clinical Medical Sciences, China-Japan Friendship Hospital, Beijing, China; ^2^Department of Pathology, Fujian Cancer Hospital, Fujian Medical University Cancer Hospital, Fuzhou, China; ^3^Department of Radiobiology, Fujian Cancer Hospital, Fujian Medical University Cancer Hospital, Fuzhou, China; ^4^Department of Cardiology, First Affiliated Hospital of Fujian Medical University, Fuzhou, China; ^5^Department of General Surgery, China-Japan Friendship Hospital, Beijing, China

**Keywords:** digestive tract cancer, neutrophil-to-lymphocyte ratio, platelet-to-lymphocyte ratio, mortality, prognosis, FIESTA study

## Abstract

**Objectives:** We sought to determine the optimal cutting points for two inflammatory biomarkers, neutrophil-to-lymphocyte ratio (NLR), and platelet-to-lymphocyte ratio (PLR), to assess their prognostic value in patients with postoperative digestive tract cancers overall and by cancer sites, and further to construct an inflammation-related index based on the two biomarkers and assess its predictive performance.

**Methods:** Total 6,865 assessable patients with digestive tract cancers who underwent tumor resection were consecutively enrolled from Fujian Cancer Hospital between January 2000 and December 2010, including 2535/3012/1318 patients with esophageal/gastric/colorectal cancer. The latest follow-up (median: 44.9 months) ended in December 2015. Optimal cutting points were determined using survival tree analysis overall and by cancer sites.

**Results:** Among all study patients, the optimal cutting points were 2.07 and 168.50 to define high and low NLR and PLR, respectively. High NLR (hazard ratio [HR]: 1.48, 95% confidence interval [CI]: 1.37–1.61) and high PLR (HR: 1.41, 95% CI: 1.29–1.53) were associated with a significantly increased risk for the mortality of digestive tract cancers as a whole. By cancer sites, effect-size estimates were comparable and statistically significant. Elevation over the selected optimal cutting points for both NLR and PLR was associated with 1.69-fold increased risk of cancer-specific mortality compared to patients with simultaneously low NLR and PLR among all study patients, and this association persisted by cancer sites, especially for gastric cancer.

**Conclusions:** Our findings demonstrate that the preoperative integrated NLR and PLR, as an inflammation-related index, is a significant independent predictor for postoperative mortality in Chinese patients with digestive tract cancers both overall and by cancer sites.

## Introduction

Digestive tract cancers are common and pose a heavy health burden in both developed and developing countries. In China, esophageal cancer (EC), gastric cancer (GC), and colorectal cancer (CRC) constitute three most frequently occurring cancers in digestive tract system, with the corresponding incidence of 477.9, 679.1, and 376.3 per 100,000 and the mortality of 375.0, 498.0, and 191.0 per 100,000, respectively in 2015 (1). Despite the advances made in multidisciplinary cancer management over the past years, a poor prognosis in patients with digestive tract cancers remains, even after receiving tumor resection (2). Currently, the debates regarding how to improve the prognosis and prolong survival time in patients with resectable digestive tract cancers are ongoing and unsettled. Identification of non-invasive and easy-to-obtain biomarkers has proven to be feasible and effective, yet no consensus has been reached, probably due to the differences in population background, sample size, follow-up interval or cancer site.

Evidence is mounting supporting an important role of systemic inflammation response in carcinogenesis (3). Further, a preoperative systemic inflammation score has been suggested as a useful indicator of postoperative survival in patients with GC (4). Of clinical biomarkers in systemic inflammation, neutrophil-to-lymphocyte ratio (NLR) and platelet-to-lymphocyte ratio (PLR) are extensively studied in the medical literature, mainly because they are easily measured, reproducible and inexpensive. In theory, the neutrophils act as cancer-promoting leukocytes, capable of stimulating tumorigenesis and suppressing anti-cancer immune response, while the host's anti-cancer immune response greatly depends on the lymphocytes (5). The platelets can provide a procoagulant surface facilitating amplification of cancer-related coagulation, and facilitate cancer growth and dissemination (6). Many clinical and epidemiological studies have examined the association of NLR and PLR with postoperative survival in patients with digestive tract cancers using different cutting points (7–10), limiting between-study comparisons. It is widely accepted that the accuracy of cutting points mainly depends on statistical power and follow-up interval.

To derive a more reliable estimate, we, in the ongoing Fujian prospective investigation of cancer (FIESTA) cohort (11–23), sought to determine the optimal cutting points for both NLR and PLR before surgery when assessing their prognostic value in patients with postoperative digestive tract cancers overall and by cancer sites. We further attempted to construct an inflammation-related index based on the integration of NLR and PLR, and assessed its predictive performance.

## Methods

### Study Patients

As we previously recorded in the FIESTA study for each type of digestive tract cancers (11–23), a total of 7,757 patients were consecutively enrolled from the Department of Thoracic Surgery, Fujian Cancer Hospital & Fujian Medical University Cancer Hospital (the former Fujian Provincial Cancer Hospital) during the period between January 2000 and December 2010. Of all study patients, there were 2,886 patients with EC who underwent three-field lymphadenectomy, 3,413 patients with GC who underwent radical gastrectomy, and 1,458 patients with CRC who underwent radical resection. The Ethics Committee of Fujian Cancer Hospital & Fujian Medical University Cancer Hospital approved the present study, and informed consents were signed by all patients.

### Tissue Collection and Diagnosis

Primary cancer and adjacent normal tissue samples were resected during the surgery and fixed in 10% neutral-buffered formalin, and further paraffin-embedded using standard procedures. All pathological assays were completed at the Department of Pathology, Fujian Cancer Hospital & Fujian Medical University Cancer Hospital.

### Follow-Up Assessment

Postoperative patients were followed up every 6–12 months by face-to-face interviews at the Out-Patient Department, Fujian Cancer Hospital & Fujian Medical University Cancer Hospital, or by phone calls or postal mails if they missed appointments. The follow-up began from initial admission after the surgery since January 2000 to the date of deaths attributable to the causes other than digestive tract cancers or the end of follow-up visits until December 31, 2015, whichever came first.

### Demographic and Clinicopathologic Characteristics

Demographic characteristics at baseline, including age (at the time of surgery), gender, smoking status (categorized as never, former and current smoking, with the latter two combined as ever smoking), drinking status (categorized as never, former and current alcohol drinking, with the latter two combined as ever drinking), and family cancer history (one or more direct relatives diagnosed with cancers except non-melanoma skin cancer within three generations) were obtained by a self-designed questionnaire after agreeing to participation.

Body weight and height were measured after removal of shoes and when wearing light clothing. Body mass index (BMI) was calculated as weight in kilograms divided by the square of height in meters. Blood pressure was measured by trained and certified examiners according to the standard protocols recommended by the American Heart Association (24).

Routine blood biomarkers, including neutrophil, lymphocyte, monocyte, eosinophil, basophil, white blood cell count, red blood cell count, hemoglobin, red cell distribution width, and platelet count were measured using fasting venous blood samples taken at the morning of receiving the surgery by the SYSMEX XE-2100 Automatic Blood Cell Analyzer (Sysmex, Kobe, Japan) at the Clinical Laboratory, Fujian Cancer Hospital & Fujian Medical University Cancer Hospital. The interval from blood drawing to clinical assays was <4 h. NLR and PLR were calculated accordingly.

Clinicopathologic characteristics were obtained from medical charts and/or pathological reports, including tumor node metastasis (TNM) stage [according to the 7th Edition of the UICC/AJCC TNM Staging system (25)], depth of invasion (T1-T4), regional lymph node metastasis (LNM) (N0-N3), distant metastasis (M0 and M1), tumor size (in centimeters) and tumor embolus.

### Statistical Analysis

Data are expressed as median (interquartile range) or number (proportion) where appropriate. Differences between two groups were compared by the Wilcoxon rank-sum or Chi-square test where appropriate. Survival rates were compared by the Kaplan-Meier curves and differences in survival time were judged by the Log-rank tests. Survival tree analysis by the STREE program (available at the website: http://c2s2.yale.edu/software/stree/) was used to determine the optimal cutting points for both NLR and PLR among all study patients and separately by three cancer sites. The survival tree algorithm can recursively split patients into two groups according to many cutoff points, and the cutoff point is optimal when the two groups have the most different Kaplan-Meier survival curves, meaning that the two groups have the minimum *p*-value for the log-rank test. Proportional hazards assumption was checked by Weighted Schoenfeld residuals. Hazard ratio (HR) and 95% confidence interval (CI) for postoperative mortality were estimated by adjusted and unadjusted Cox proportional hazard models. In addition, permutation testing using 1,000 bootstrap replications was performed to internally validate the results. Predictive accuracy of the basic model gained by adding integrated NLR and PLR (namely INP) was appraised from both calibration and discrimination aspects. Specifically, calibration statistics included Akaike information criterion (AIC) and Bayesian information criterion (BIC), as well as the −2 log likelihood ratio test and the area under the receiver operating characteristic (ROC) curve (AUC) to assess how closely the prediction probability for the addition of INP reflected the actual observed risk and the global fit of modified risk model. Discrimination statistics included net reclassification improvement (NRI) and integrated discrimination improvement (IDI) (26, 27) to justify the improvement in prediction performance, as well as the Harrell's C index to inspect whether the addition of INP to the basic model can differentiate among patients who died or survived.

All statistical tests were two-sided, and *p* < 0.05 was considered to be statistically significant. SAS software, version 9.4 (SAS Institute Inc.) and STATA software, version 14.1 (Stata Corp, College Station, TX) were adopted to complete statistical analyses, unless otherwise indicated.

## Results

### Baseline Characteristics

After removing patients with incomplete data and dying from causes other than digestive tract cancers as we previously reported (11–23), 6,865 patients were assessable in the current analysis, including 2,535 patients with EC, 3,012 patients with GC, and 1,318 patients with CRC. The follow-up time ranged from 1.0 month to 188.9 months (median: 44.9 months). Total 2,808 deaths occurred during the follow-up, including 1,065 patients with EC, 1,331 patients with GC, and 412 patients with CRC. Baseline characteristics differed significantly except BMI and family cancer history between non-survivors (*n* = 2,808) and survivors (*n* = 4,057) (Supplementary Table 1).

### Cutting Point Determination

Weighted Schoenfeld residuals did not indicate major departures from the proportional hazards assumption, and so Cox proportional hazard model was employed. As continuous variables, after adjusting for age, gender, smoking, drinking, BMI and family cancer history, per standard deviation increments (NLR: 2.48 and PLR: 94.68) in preoperative NLR (HR: 1.07, 95% CI: 1.05–1.10, *p* < 0.001) and PLR (HR: 1.07, 95% CI: 1.05–1.09, *p* < 0.001) were significantly associated with poor survival of digestive tract cancers as a whole.

To determine the optimal cutting points for NLR and PLR among all study patients and in patients separately with EC, GC, and CRC, we adopted the survival tree analysis, and found that the optimal cutting points for NLR and PLR were 2.07 and 168.50 among all study patients, 2.36 and 159.23 in patients with EC, 1.97 and 188.0 in patients with GC and 3.37 and 264.29 for patients with CRC, respectively. The optimal cutting points selected can split patients into two groups with the maximal difference in survival time. The estimates of predictive performance of selected optimal cutting points for NLR and PLR in predicting cancer-specific mortality are presented in Supplementary Table 2. Among all study patients, the cutting points selected has sensitivity and specificity of 77.68 and 75.68% for NLR, and of 84.21 and 73.89% for PLR, respectively, and the corresponding AUC was 0.743 (95% CI: 0.728–0.757) and 0.715 (95% CI: 0.701–0.730). By cancer sites, patients with CRC had the best sensitivity, specificity, and AUC, followed by patients with gastric cancer.

In patients with digestive tract cancers overall and by cancer sites, those with NLR or PLR greater than selected cutting points were classified as high NLR or high PLR group, whereas those with NLR or PLR less than or equal to cutting points were classified as low NLR or low PLR group. After adjusting for age, gender, smoking, drinking, BMI and family cancer history, high NLR, and high PLR were significantly associated with 1.48-fold (95% CI: 1.37–1.61, *p* < 0.001) and 1.41-fold (95% CI: 1.29–1.53, *p* < 0.001) increased mortality risk of digestive tract cancers as a whole relative to low NLR and low PLR among all study patients, 1.32-fold (95% CI: 1.16–1.50, *p* < 0.001) and 1.39-fold (95% CI: 1.21–1.60, *p* < 0.001) increased risk in patients with EC, 1.83-fold (95% CI: 1.62–2.07, *p* < 0.001) and 1.58-fold increased risk in patients with GC, and 1.89-fold (95% CI: 1.50–2.38, *p* < 0.001) and 1.82-fold (1.35–2.43, *p* < 0.001) increased risk in patients with CRC, respectively (Table 1).

**Table 1:** Risk prediction of NLR and PLR as categorical variables for cancer-specific mortality in patients with postoperative digestive tract cancers overall and by cancer sites.

**Biomarkers**	**Overall**			**Esophageal cancer**			**Gastric cancer**			**Colorectal cancer**		
	***n***	**HR (95% CI)**	p^*^	***n***	**HR (95% CI)**	p^*^	***n***	**HR (95% CI)**	p^*^	***n***	**HR (95% CI)**	p^*^
**NLR**												
Low NLR	3,387	Reference		1,598	Reference		1,288	Reference		1,031	Reference	
High NLR	3,108	1.48 (1.37–1.61)	<0.001	893	1.32 (1.16–1.50)	<0.001	1,469	1.83 (1.62–2.07)	<0.001	216	1.89 (1.50–2.38)	<0.001
**PLR**												
Low PLR	4,555	Reference		1,832	Reference		2,018	Reference		1,132	Reference	
High PLR	1,911	1.41 (1.29–1.53)	<0.001	637	1.39 (1.21–1.60)	<0.001	734	1.58 (1.40–1.80)	<0.001	113	1.82 (1.35–2.43)	<0.001

*NLR, neutrophil-to-lymphocyte ratio; PLR, platelet-to-lymphocyte ratio; HR, hazard ratio; 95% CI, 95% confidence interval. Effect-size estimates were calculated under the COX proportional hazards regression models*.

* *p was adjusted for age, gender, smoking, drinking, body mass index, and family cancer history. Cutting points: 2.07 for NLR and 168.50 for PLR among all study patients; 2.36 for NLR and 159.23 for PLR in patients with esophageal cancer; 1.97 for NLR and 188.0 for PLR in patients with gastric cancer; 3.37 for NLR and 264.29 for PLR in patients with colorectal cancer*.

Further subgroup analyses were conducted according to clinicopathologic characteristics, and high NLR and high PLR were found to be associated with significantly high risk for cancer-specific mortality within each subgroup among all study patients except high PLR in invasion depth T1/T2 group (Table 2). Subgroup analyses in patients with GC revealed that only patients with positive distant metastasis showed a non-significant association between high PLR, high NLR and cancer-specific mortality (Table 2).

**Table 2:** Stratified risk prediction of NLR and PLR as categorical variables for cancer-specific mortality in patients with postoperative digestive tract cancers overall and by cancer sites.

**Biomarkers**	**Overall**			**Esophageal cancer**			**Gastric cancer**			**Colorectal cancer**		
	**n**	**HR (95% CI)**	p^*^	**n**	**HR (95% CI)**	p^*^	**n**	**HR (95% CI)**	p^*^	**n**	**HR (95% CI)**	p^*^
**NLR**												
**Tumor-node-metastasis stage**												
I/II	1,006	1.46 (1.20–1.77)	<0.001	311	1.11 (0.83–1.48)	0.495	306	2.33 (1.51–3.59)	<0.001	104	2.64 (1.74–4.03)	<0.001
III/IV	2,073	1.39 (1.28–1.52)	<0.001	570	1.27 (1.10–1.46)	0.001	1156	1.53 (1.34–1.74)	<0.001	109	1.77 (1.33–2.36)	<0.001
**Invasion depth**												
T1/T2	514	1.35 (1.06–1.72)	0.016	180	1.07 (0.77–1.50)	0.693	181	2.34 (1.32–4.13)	0.004	32	1.90 (0.86–4.20)	0.115
T3/T4	2,563	1.40 (1.28–1.52)	<0.001	695	1.30 (1.13–1.50)	<0.001	1283	1.57 (1.38–1.79)	<0.001	183	1.82 (1.42–2.32)	<0.001
**Regional lymph node metastasis**												
N0	1,060	1.50 (1.24–1.80)	<0.001	336	1.16 (0.89–1.53)	0.275	318	2.13 (1.42–3.19)	<0.001	115	2.51 (1.71–3.67)	<0.001
N1/N2/N3	1,741	1.42 (1.29–1.56)	<0.001	445	1.30 (1.10–1.54)	0.002	977	1.54 (1.34–1.77)	<0.001	100	1.77 (1.31–2.39)	<0.001
**Distant metastasis**												
Negative	2,178	1.52 (1.37–1.70)	<0.001	377	1.22 (0.96–1.56)	0.108	1236	1.75 (1.52–2.03)	<0.001	188	1.85 (1.42–2.41)	<0.001
Positive	896	1.37 (1.21–1.54)	<0.001	498	1.30 (1.12–1.52)	<0.001	227	1.29 (1.00–1.66)	0.052	25	1.60 (0.95–2.72)	0.080
**Tumor embolus**												
Negative	2,054	1.46 (1.32–1.62)	<0.001	741	1.31 (1.13–1.52)	<0.001	839	1.93 (1.62–2.3)	<0.001	111	2.41 (1.67–3.47)	<0.001
Positive	859	1.47 (1.29–1.69)	<0.001	152	1.35 (1.04–1.76)	0.024	619	1.58 (1.32–1.89)	<0.001	39	2.58 (1.62–4.10)	<0.001
**PLR**												
**Tumor-node-metastasis stage**												
I/II	572	1.30 (1.05–1.61)	0.016	224	1.12 (0.81–1.53)	0.498	141	2.16 (1.38–3.38)	<0.001	55	1.90 (1.09–3.31)	0.024
III/IV	1321	1.27 (1.16–1.4)	<0.001	402	1.37 (1.17–1.60)	<0.001	588	1.34 (1.17–1.53)	<0.001	57	2.28 (1.61–3.23)	<0.001
**Invasion depth**												
T1/T2	274	1.31 (0.98–1.76)	0.064	136	1.09 (0.75–1.59)	0.636	74	3.44 (1.90–6.22)	<0.001	14	1.32 (0.40–4.39)	0.651
T3/T4	1,621	1.29 (1.18–1.41)	<0.001	487	1.37 (1.18–1.59)	<0.001	658	1.34 (1.17–1.52)	<0.001	98	1.83 (1.35–2.48)	<0.001
**Regional lymph node metastasis**												
N0	604	1.35 (1.10–1.64)	0.003	227	1.19 (0.88–1.60)	0.265	151	1.97 (1.30–2.99)	0.002	57	2.00 (1.20–3.32)	0.007
N1/N2/N3	1,133	1.31 (1.18–1.45)	<0.001	342	1.36 (1.14–1.62)	<0.001	493	1.38 (1.19–1.6)	<0.001	55	2.07 (1.44–2.98)	<0.001
**Distant metastasis**												
Negative	1,381	1.43 (1.28–1.60)	<0.001	259	1.28 (0.98–1.68)	0.065	590	1.48 (1.27–1.72)	<0.001	94	1.70 (1.20–2.4)	0.003
Positive	511	1.45 (1.27–1.65)	<0.001	364	1.34 (1.14–1.57)	<0.001	141	1.06 (0.83–1.34)	0.654	18	1.40 (0.76–2.59)	0.286
**Tumor embolus**												
Negative	1,238	1.45 (1.30–1.62)	<0.001	530	1.39 (1.19–1.63)	<0.001	421	1.71 (1.43–2.04)	<0.001	58	2.21 (1.41–3.47)	<0.001
Positive	553	1.36 (1.18–1.56)	<0.001	107	1.43 (1.08–1.89)	0.014	308	1.43 (1.19–1.72)	<0.001	24	2.27 (1.31–3.93)	0.003

*NLR, neutrophil-to-lymphocyte ratio; PLR, platelet-to-lymphocyte ratio; HR, hazard ratio; 95% CI, 95% confidence interval. Effect-size estimates were calculated under the COX proportional hazards regression models*.

* *p was adjusted for age, gender, smoking, drinking, body mass index, family cancer history*.

### Integrated NLR and PLR in Predicting Cancer-Specific Mortality

On a continuous scale, a three-dimension surface was plotted to show joint increments in preoperative NLR and PLR in predicting the mortality risk of digestive tract cancers as a whole (Supplementary Figure 1).

**Figure 1:**
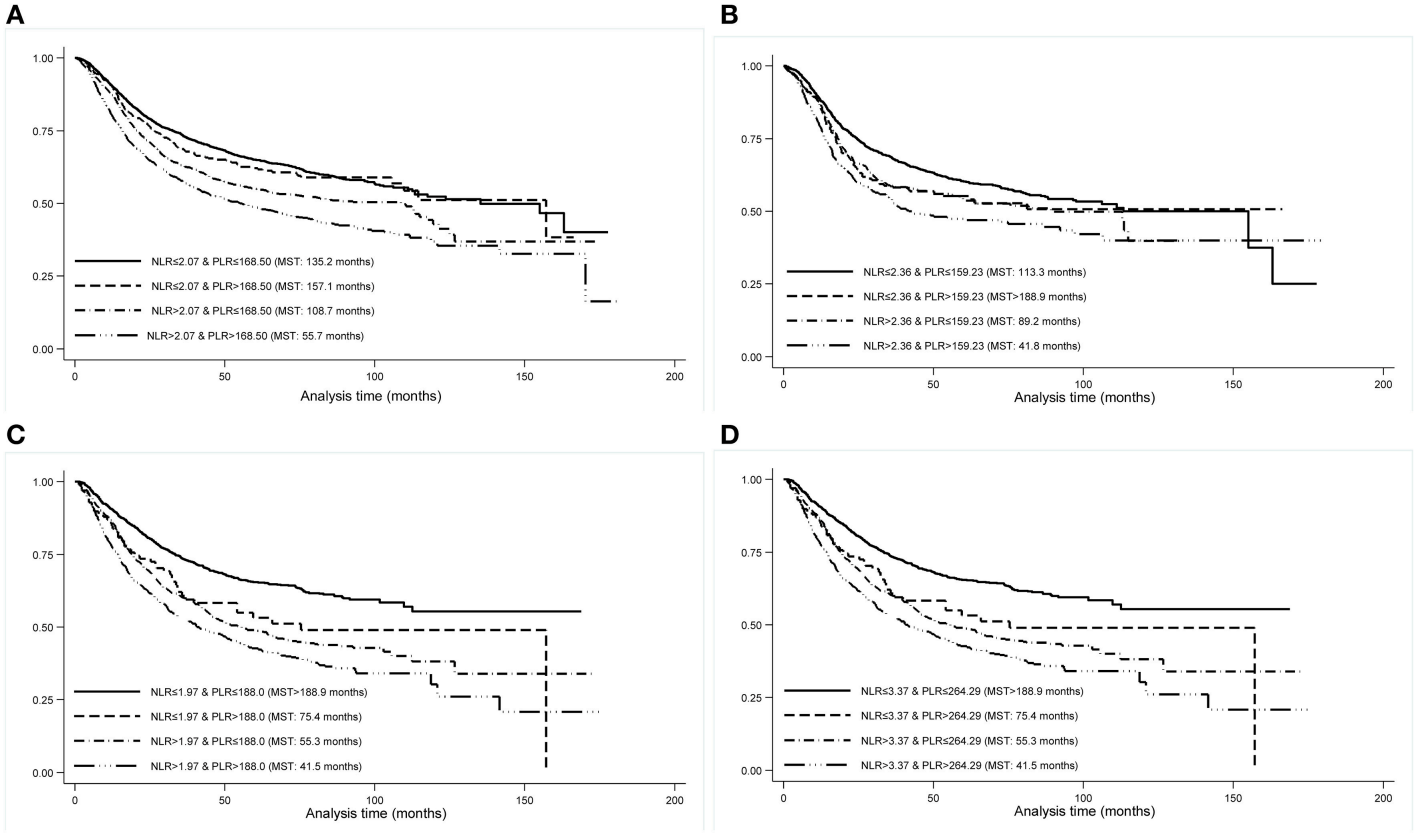
Kaplan-Meier curves by INP groups in all study patients **(A)**, patients with esophageal cancer **(B)**, patients with gastric cancer **(C)**, and patients with colorectal cancer **(D)**. INP, integrated neutrophil-to-lymphocyte ratio and platelet-to-lymphocyte ratio; MST, median survival time.

As correlation analysis indicated a strong positive relation between NLR and PLR (*r* = 0.60, *p* < 0.001) among all study patients, we generated the integrated NLR and PLR, namely INP, according to selected cutting points both overall and by cancer sites, which was defined as follows: patients with neither elevated NLR (≤ cutting point) nor PLR (≤ cutting point) were assigned a score of 0; patients with only elevated PLR (> cutting point) were assigned a score of 1; patients with only elevated NLR (> cutting point) were assigned a score of 2; patients with both elevated NLR (>cutting point) and PLR (> cutting point) were assigned a score of 3. For INP ranging from 0 to 3, there were 3,369 (49.08%), 388 (5.65%), 1,585 (23.09%), and 1,523 (22.18%) patients, respectively. The median survival time for patients with INP equal to 3 (55.7 months, Log-rank test *p* < 0.001) was significantly shorter than the other three INP groups (Figure 1), and HRs of cancer-specific mortality for patients with INP equal to 1, 2, and 3 were 1.06 (95% CI: 0.88–1.27), 1.32 (95% CI: 1.19–1.46), and 1.69 (95% CI: 1.53–1.86) relative to INP equal to 0 among all study patients, respectively. In patients with GC, INP equal to 1 (HR: 1.49, 95% CI: 1.07–2.08), 2 (HR: 1.73, 95% CI: 1.50–2.00), and 3 (HR: 2.10, 95% CI: 1.81–2.45) were associated with significantly increased risk of cancer-specific mortality. The risk for INP equal to three was 1.82 times as high as the sum of the risk in INP equal to 1 and 2 (synergy index: 1.82, 95% CI: 0.97–3.39). Relative excess risk due to the additive interaction between NLR and PLR was 0.31 (95% CI: 0.06–0.56), and the additive interaction accounted for 18% of mortality in patients with both risk factors (attributable proportion due to interaction: 0.18, 95% CI: 0.04–0.33) (Table 3).

**Table 3:** Risk prediction of INP for cancer-specific mortality in patients with postoperative digestive tract cancers overall and by cancer sites.

**INP**	**Overall**			**Esophageal cancer**			**Gastric cancer**			**Colorectal cancer**		
	**n**	**HR (95% CI)**	p^*^	**n**	**HR (95% CI)**	p^*^	**n**	**HR (95% CI)**	p^*^	**n**	**HR (95% CI)**	p^*^
INP 0	2,992	Reference		1,412	Reference		1,188	Reference		991	Reference	
INP 1	388	1.06 (0.88–1.27)	0.535	180	1.27 (1.00–1.63)	0.051	99	1.49 (1.07–2.08)	0.020	38	1.38 (0.82–2.33)	0.224
INP 2	1,558	1.32 (1.19–1.46)	<0.001	418	1.19 (1.01–1.42)	0.043	828	1.73 (1.50–2.00)	<0.001	140	1.73 (1.30–2.29)	<0.001
INP 3	1,523	1.69 (1.53–1.86)	<0.001	457	1.52 (1.30–1.78)	<0.001	635	2.10 (1.81–2.45)	<0.001	75	2.36 (1.67–3.34)	<0.001
**PERMUTATION TESTING USING 1,000 BOOTSTRAP REPLICATIONS**												
INP 0	2,992	Reference			Reference			Reference			Reference	
INP 1	388	1.06 (0.87–1.29)	0.558		1.27 (0.98–1.66)	0.072		1.49 (1.05–2.11)	0.025		1.38 (0.79–2.42)	0.258
INP 2	1,558	1.32 (1.20–1.46)	<0.001		1.19 (1.01–1.41)	0.035		1.73 (1.51–1.99)	<0.001		1.73 (1.30–2.30)	<0.001
INP 3	1,523	1.69 (1.54–1.85)	<0.001		1.52 (1.26–1.84)	<0.001		2.10 (1.82–2.44)	<0.001		2.36 (1.64–3.43)	<0.001
RERI	0.31 (0.06–0.56)		0.05 (−0.35–0.46)		−0.12 (−0.68–0.45)		0.26 (−0.88–1.39)	
AP	0.18 (0.04–0.33)		0.04 (−0.23–0.30)		−0.06 (−0.33–0.22)		0.11 (−0.35–0.56)	
SI	1.82 (0.97–3.39)		1.11 (0.48–2.59)		0.91 (0.57–1.45)		1.23 (0.48–3.16)	

*NLR, neutrophil-to-lymphocyte ratio; PLR, platelet-to-lymphocyte ratio; INP, integrated NLR and PLR; HR, hazard ratio; 95% CI, 95% confidence interval; RERI, relative excess risk of interaction; AP, attributable proportion; SI, synergy index. Effect-size estimates were calculated under the COX proportional hazards regression models*.

* *p was adjusted for age, gender, smoking, drinking, body mass index and family cancer history. Cutting points: 2.07 for NLR and 168.50 for PLR among all study patients; 2.36 for NLR and 159.23 for PLR in patients with esophageal cancer; 1.97 for NLR and 188.0 for PLR in patients with gastric cancer; 3.37 for NLR and 264.29 for PLR in patients with colorectal cancer. Definitions: INP 0: NLR ≤ cutting point and PLR ≤ cutting point; INP 1: NLR ≤ cutting point and PLR > cutting point; INP 2: NLR > cutting point and PLR ≤ cutting point; INP 3: NLR > cutting point and PLR > cutting point*.

Additionally, for the risk prediction of INP for cancer-specific mortality, robust permutation testing was performed using 1,000 bootstrap replications subsequently (Table 3), and no change in significance level was detected.

The predictive accuracy of the basic model (including age, gender, smoking, drinking, BMI, family cancer history, TNM stage, and tumor embolus) with and without INP for postoperative digestive tract cancers overall and by cancer sites is presented in Supplementary Table 3. Among all study patients and in patients separately with EC, GC, and CRC, adding INP to the basic model produced small AIC and BIC statistics, and likelihood ratio test indicated that INP was indeed a part of true model and carried a better fit. The AUC differed significantly between the basic model with and without adding INP. Moreover, the probabilities of NRI and IDI were statistically significant after adding INP to the basic model. Harrell's C index showed that the basic model with and without INP was well-performed.

Patients with both evaluated NLR and PLR showed higher risk for cancer-specific mortality in all subgroups stratified by clinicopathologic characteristics among all study patients and in patients by three cancer sites (Supplementary Table 4).

## Discussion

Via a comprehensive analysis of the long-term FIESTA cohort, we identified the optimal cutting points for two inflammatory biomarkers, NLR and PLR, in 6,865 patients with digestive tract cancers overall and by cancer sites. Importantly, we have generated an inflammation-related index based on the integrated NLR and PLR, namely INP, and found that this index exhibited better performance of survival prediction for cancer-specific mortality in Chinese patients with digestive tract cancers overall and by cancer sites. The findings of this study will advance our understanding on the clinical relevance of NLR and PLR, as well as their integration form in the development and progression of digestive tract cancers.

It is widely recognized that inflammation plays a contributory role in the initiation, progression and prognosis of various types of cancers, especially in digestive tract system (3, 28–30). Several systemic inflammation-based prognostic biomarkers have been identified, such as NLR, PLR, lymphocyte-to-monocyte ratio and C-reactive protein, as potential cancer risk or prognostic factors (31–35). In particular, NLR and PLR are two inflammatory biomarkers that are extensively evaluated in the medical literature, and they were found to be associated with the significant risk of EC, GC, and CRC in our prior studies (13, 15, 16). However, a common problem facing scientific community is to seek optimal cutting points for both biomarkers, which are constrained by some methodological issues, such as statistical power and follow-up interval.

The majority of prior studies have employed the receiver operating characteristic (ROC) curve or quantile to determine optimal cutting point. These cutting points are heterogeneous across studies, even for the same type of cancer or at the same place (31, 36–41). A lack of sufficient power has been cited as a major reason for inconsistencies. Several splitting criteria have been developed, such as classification and regression trees (CART) and multivariate adaptive regression splines (MARS) (42, 43). Although the relative merits of these criteria are not clearly resolved, survival tree-based method has been applicable to more general situations based on scientific judgement (44). Therefore, we employed survival tree analysis to determine the optimal cutting points in predicting the cancer-specific mortality postoperatively, and further performed validation in patients with digestive tract cancer overall and by cancer sites. Using the derived optimal cutting points among all study patients, we found that both high NLR and high PLR were associated with an ~1.5-fold increased risk of cancer-specific mortality in the present study, and this association persisted for three types of digestive tract cancers, especially GC.

Cancer is a highly complex family of diseases, to which multiple factors contribute interactively, and so the contribution of any single biomarker, by itself, might be small and depends on the others. Given this fact, we thereby, on the basis of the integration of NLR and PLR at their optimal cutting points, developed the INP, as an inflammation-related index to assess its association with the risk of digestive tract cancer-specific mortality overall and by cancer sites. Although the integration of NLR and PLR as a composite biomarker has been widely investigated, comparisons between the results of different studies are difficult due to diverse cutting points selected (38, 45). For instance, Tao and colleagues found a strong predictive effect for combined NLR-PLR index in 153 patients with CRC who received adjuvant chemotherapy (46). Feng et al. also found that INP was an independent prognostic marker in patients with EC without neoadjuvant or adjuvant treatment (47). By contract in this present study, on a binary scale we found that the effect of NLR on prognosis was greater than that of PLR in terms of hazard ratio, and the parameter INP was an independent predictor, with high NLR and high PLR together predicting poor postsurgical survival. Although broad replication offers valuable information for a better understanding of NLR and PLR in cancer survival, the exact mechanisms are elusive currently. It is possible that platelet can regulate immune response, inflammation and angiogenesis, in cooperation with neutrophils and lymphocytes (48). Activated platelets promote cancer metastasis and angiogenesis via releasing various cytokines and forming cancer embolus, so that it can escape from the immunocyte (49, 50). Moreover, platelet activation can trigger platelet-neutrophils interaction, alter the immunocyte subpopulations and enhance the differentiation and cytokine production of T-effector cell (51–53).

There were several potential limitations for the present study. Firstly, this study was performed in a single hospital, which restricted the generalizability, although it can facilitate consistency of evaluation. Additionally, external validation is necessary. Secondly, due to the difficulty in identifying an external group, we are unable to validate our findings in an independent population. Thirdly, only cancer-specific mortality was analyzed in this study, because information on deaths from causes other than digestive tract cancers is incomplete, which precludes further competing risk analysis. Fourthly, patients were exclusively enrolled from a southern city in China, which restricted racial or ethnical extrapolation. Fifthly, the recruitment period was as long as 10 years, during which the advances in surgical therapies might introduce a possible bias and impact the prognosis of patients due to time effect.

Taken together, our findings indicate that preoperative INP, as an inflammation-related index, is a significant independent predictor for postoperative cancer-specific mortality in patients with digestive tract cancers overall and by cancer sites in Chinese. For practical reasons, data from this study may provide basic evidence that patients with digestive tract cancers especially GC who have elevated INP based on the optimal cutting points of NLR and PLR, presumably need close monitoring for prolonging survival and improving quality of life after the surgery, and are thus of significant clinical value.

## Ethics Statement

All procedures followed were in accordance with the ethical standards of the responsible committee on human experimentation and with the Helsinki Declaration of 1964 and later versions. Informed consent to be included in the study, or the equivalent, was obtained from all patients.

## Author Contributions

DH, FP, and WN planned and designed the study, and directed its implementation. FP, DH, JL, and XZhe drafted the protocol. DH, XL, HZ, and YX obtained statutory and ethics approvals. DH, XL, HZ, and YX contributed to data acquisition. XZha, JJ, and WN conducted statistical analyses. WN, DH, FP, JJ, JL, and XZhe had access to all raw data. XZha, FP, DH, and WN did the data preparation and quality control. XZha, FP, JJ, and WN wrote and revised the manuscript. All authors read and approved the final manuscript prior to submission.

### Conflict of Interest Statement

The authors declare that the research was conducted in the absence of any commercial or financial relationships that could be construed as a potential conflict of interest.
