# Supplementary material for: Prognostic Value of an Inflammation-Related Index in 6,865 Chinese Patients With Postoperative Digestive Tract Cancers: The FIESTA Study
Source: Front Oncol. 2019 May 22;9:427. doi: 10.3389/fonc.2019.00427 (PMC6538942; doi:10.3389/fonc.2019.00427)

Supplementary Table 1. Baseline characteristics of patients with digestive tract malignancies.

Baseline characteristics		Non-survivors (n=2808)	Survivors (n=4057)	p^*
Age (years)		57.00 (50.00-65.00)	58.00 (50.00-66.00)	<0.001
Males		2643 (70.52)	1988 (74.32)	<0.001
Smoking		933 (24.89)	763 (28.52)	0.001
Drinking		360 (9.61)	323 (12.07)	0.002
Body mass index (kg/m^2)		22.41 (20.48-24.46)	22.46 (20.43-24.91)	0.183
Family cancer history		396 (10.58)	284 (10.63)	0.995
Tumor-node-metastasis stage				
	I	639 (18.30)	41 (1.65)	<0.001
	II	1125 (32.23)	353 (14.19)	
	III	1684 (48.24)	1627 (65.42)	
	IV	43 (1.23)	466 (18.74)	
Invasion depth				
	T1	485 (13.89)	46 (1.85)	<0.001
	T2	572 (16.38)	217 (8.73)	
	T3	1841 (52.74)	1393 (56.01)	
	T4	593 (16.99)	831 (33.41)	
Regional lymph node metastasis				
	N0	1713 (49.07)	429 (17.25)	<0.001
	N1	996 (28.53)	762 (30.64)	
	N2	677 (19.39)	931 (37.43)	
	N3	105 (3.01)	365 (14.68)	
Distant metastasis				
	Negative	2934 (84.04)	1397 (56.17)	<0.001

	Positive	557 (15.96)	1090 (43.83)	
Tumor embolus				
	Negative	2773 (79.43)	1526 (61.36)	<0.001
	Positive	718 (20.57)	961 (38.64)	
Tumor size (cm)		4.00 (3.00-6.00)	5.00 (4.00-7.00)	<0.001
Number of regional lymph node metastasis		0 (0-3.00)	4.00 (1.00-8.00)	<0.001

Data are expressed as median (interquartile range) or count (percentage) where appropriate. $^{*}p$ was calculated by the Wilcoxon rank-sum test for continuous variables and the Chi-square test for categorical variables.

Supplementary Table 2. Estimates of predictive performance of selected optimal cutting points for neutrophil-to-lymphocyte ratio and platelet-to-lymphocyte ratio in predicting cancer-specific mortality.

**Cancer type**	**Neutrophil-to-lymphocyte ratio**			**Platelet-to-lymphocyte ratio**		
	**Sensitivity**	**Specificity**	**AUC (95% CI)**	**Sensitivity**	**Specificity**	**AUC (95% CI)**
Overall	77.68%	75.68%	0.743 (0.728, 0.757)	84.21%	73.89%	0.715 (0.701, 0.730)
Esophageal cancer	73.25%	73.67%	0.677 (0.654, 0.699)	77.75%	67.64%	0.671 (0.648, 0.694)
Gastric cancer	75.15%	78.84%	0.775 (0.754, 0.796)	78.60%	72.00%	0.736 (0.715, 0.758)
Colorectal cancer	86.70%	79.96%	0.793 (0.758, 0.828)	92.99%	77.36%	0.748 (0.712, 0.783)

Abbreviations: AUC, area under the receiver operating characteristic curve; 95% CI, 95% confidence interval. Selected cutting points by survival tree analysis: 2.07 for NLR and 168.50 for PLR in overall patients; 2.36 for NLR and 159.23 for PLR in patients with esophageal cancer; 1.97 for NLR and 188.0 for PLR in patients with gastric cancer; 3.37 for NLR and 264.29 for PLR in patients with colorectal cancer.

Supplementary Table 3. Predictive accuracy of basic model with and without INP for postoperative digestive tract cancers overall and by cancer sites.

Predictive measurement	Overall	Esophageal cancer	Gastric cancer	Colorectal cancer
Calibration statistics				
AIC: basic model	41859	14894	16530	5234
AIC: basic model + INP	40793	14390	16078	5178
BIC: basic model	41899	14928	16565	5265
BIC: basic model + INP	40840	14430	16119	5214
LR test: χ^2	112.56	24.39	104.44	29.74
LR test: *p*	<0.001	<0.001	<0.001	<0.001
AUC: basic model	0.647 (0.632, 0.662)	0.677 (0.653, 0.700)	0.653 (0.630, 0.676)	0.651 (0.617, 0.686)
AUC: basic model + INP	0.689 (0.675, 0.704)	0.693 (0.670, 0.717)	0.727 (0.705, 0.749)	0.701 (0.667, 0.736)
AUC comparison: *p*	<0.001	0.022	<0.001	0.002
Discrimination statistics				
NRI: estimate	0.073	0.047	0.118	0.123
NRI: *p*	<0.001	0.012	<0.001	<0.001
IDI: estimate	0.017	0.008	0.039	0.025
IDI: *p*	<0.001	<0.001	<0.001	<0.001
Harrell's C index	0.580	0.583	0.306	0.585

Abbreviations: INP, integrated NLR and PLR; AIC, Akaike information criterion; BIC, Bayesian information criteria; LR test, likelihood ratio test; AUC, area under the receiver operating characteristic curve; NRI, net reclassification improvement; IDI, integrated discrimination improvement. Basic model included age, gender, smoking, drinking, body mass index, family cancer history, tumor-node-metastasis stage and tumor embolus.

Supplementary Table 4. Risk prediction of INP for cancer-specific mortality stratified by clinicopathologic characteristics in patients with postoperative digestive tract cancers overall and by cancer sites.

**INP**	**Overall**			**Esophageal cancer**			**Gastric cancer**			**Colorectal cancer**		
	**n**	**HR (95% CI)**	***p*** *	**n**	**HR (95% CI)**	***p*** *	**n**	**HR (95% CI)**	***p*** *	**n**	**HR (95% CI)**	***p*** *
Tumor-node-metastasis stage												
Stage I/II												
INP 0	1266	Reference		651	Reference		412	Reference		494	Reference	
INP 1	126	0.68 (0.39-1.20)	0.183	71	1.08 (0.63-1.84)	0.787	27	1.04 (0.25-4.37)	0.952	21	0.98 (0.30-3.13)	0.968
INP 2	552	1.25 (0.99-1.59)	0.066	152	1.05 (0.72-1.55)	0.784	192	1.87 (1.13-3.10)	0.015	69	2.49 (1.52-4.10)	<0.001
INP 3	446	1.61 (1.27-2.05)	<0.001	153	1.15 (0.79-1.67)	0.473	114	3.22 (1.91-5.42)	<0.001	34	3.23 (1.72-6.05)	<0.001
Stage III/IV												
INP 0	1709	Reference		745	Reference		773	Reference		492	Reference	
INP 1	258	1.01 (0.83-1.22)	0.951	107	1.26 (0.96-1.67)	0.101	71	1.44 (1.01-2.04)	0.042	16	2.31 (1.28-4.15)	0.005
INP 2	991	1.29 (1.16-1.44)	<0.001	263	1.14 (0.94-1.39)	0.176	633	1.52 (1.31-1.78)	<0.001	68	1.56 (1.10-2.22)	0.013
INP 3	1063	1.51 (1.35-1.67)	<0.001	295	1.47 (1.23-1.76)	<0.001	517	1.65 (1.41-1.93)	<0.001	41	2.48 (1.63-3.78)	<0.001
Invasion depth												
T1/T2												
INP 0	837	Reference		465	Reference		295	Reference		190	Reference	
INP 1	71	0.78 (0.40-1.54)	0.481	54	0.96 (0.54-1.71)	0.879	17	2.04 (0.47-8.79)	0.340	8	1.88 (0.43-8.13)	0.400
INP 2	305	1.17 (0.87-1.58)	0.295	93	0.96 (0.62-1.48)	0.843	124	1.58 (0.78-3.22)	0.202	25	2.54 (1.09-5.93)	0.032
INP 3	203	1.58 (1.15-2.19)	0.005	82	1.17 (0.74-1.85)	0.510	57	4.68 (2.35-9.31)	<0.001	6	1.19 (0.16-9.08)	0.868
T3/T4												
INP 0	2137	Reference		928	Reference		891	Reference		797	Reference	

INP 1	314	1.00 (0.83-1.22)	0.963	124	1.33 (1.01-1.75)	0.041	82	1.34 (0.95-1.89)	0.098	29	1.42 (0.81-2.49)	0.223
INP 2	1235	1.27 (1.14-1.42)	<0.001	319	1.21 (1.00-1.46)	0.050	701	1.56 (1.34-1.81)	<0.001	114	1.62 (1.20-2.19)	0.002
INP 3	1307	1.52 (1.37-1.68)	<0.001	363	1.48 (1.24-1.76)	<0.001	576	1.68 (1.44-1.96)	<0.001	69	2.26 (1.59-3.22)	<0.001
Regional lymph node metastasis												
N0												
INP 0	1221	Reference		626	Reference		388	Reference		511	Reference	
INP 1	126	0.94 (0.59-1.51)	0.813	63	1.33 (0.79-2.25)	0.281	28	1.94 (0.69-5.50)	0.210	20	0.93 (0.29-2.97)	0.905
INP 2	575	1.35 (1.08-1.70)	0.009	169	1.22 (0.86-1.73)	0.276	194	1.89 (1.18-3.05)	0.009	77	2.26 (1.44-3.55)	<0.001
INP 3	478	1.65 (1.31-2.07)	<0.001	164	1.20 (0.85-1.71)	0.302	123	2.71 (1.65-4.47)	<0.001	37	3.28 (1.87-5.76)	<0.001
N1/N2/N3												
INP 0	1610	Reference		658	Reference		750	Reference		476	Reference	
INP 1	237	1.01 (0.82-1.24)	0.946	102	1.12 (0.82-1.51)	0.483	63	1.42 (0.97-2.07)	0.068	17	1.97 (1.10-3.53)	0.024
INP 2	828	1.29 (1.14-1.46)	<0.001	193	1.11 (0.88-1.40)	0.366	542	1.50 (1.27-1.77)	<0.001	62	1.59 (1.10-2.30)	0.014
INP 3	896	1.56 (1.39-1.75)	<0.001	240	1.53 (1.24-1.87)	<0.001	430	1.68 (1.42-2.00)	<0.001	38	2.35 (1.50-3.67)	<0.001
Distant metastasis												
Negative												
INP 0	2243	Reference		713	Reference		1083	Reference		927	Reference	
INP 1	302	1.18 (0.94-1.49)	0.149	76	1.43 (0.90-2.26)	0.128	87	1.72 (1.18-2.49)	0.004	34	1.47 (0.82-2.64)	0.195
INP 2	1086	1.40 (1.23-1.61)	<0.001	188	1.27 (0.92-1.74)	0.144	729	1.75 (1.48-2.07)	<0.001	127	1.80 (1.31-2.46)	<0.001
INP 3	1079	1.73 (1.52-1.97)	<0.001	183	1.33 (0.97-1.82)	0.079	503	1.93 (1.61-2.31)	<0.001	60	2.11 (1.39-3.20)	<0.001
Positive												
INP 0	730	Reference		680	Reference		103	Reference		59	Reference	

INP 1	83	0.98 (0.73-1.34)	0.923	102	1.07 (0.80-1.44)	0.635	12	0.60 (0.27-1.31)	0.198	3	1.37 (0.40-4.63)	0.616
INP 2	454	1.14 (0.97-1.32)	0.105	224	1.10 (0.90-1.36)	0.348	96	1.25 (0.91-1.71)	0.172	10	1.81 (0.86-3.83)	0.120
INP 3	428	1.65 (1.42-1.92)	<0.001	262	1.51 (1.25-1.82)	<0.001	129	1.24 (0.93-1.65)	0.146	15	1.51 (0.77-2.98)	0.232
Tumor embolus												
Negative												
INP 0	2108	Reference		1183	Reference		771	Reference		536	Reference	
INP 1	248	1.18 (0.94-1.50)	0.160	147	1.29 (0.97-1.70)	0.077	59	1.73 (1.08-2.78)	0.023	21	1.44 (0.63-3.30)	0.387
INP 2	1046	1.31 (1.15-1.49)	<0.001	345	1.19 (0.98-1.45)	0.079	474	1.82 (1.48-2.25)	<0.001	74	2.07 (1.32-3.25)	0.001
INP 3	990	1.70 (1.50-1.93)	<0.001	383	1.51 (1.26-1.82)	<0.001	362	2.28 (1.84-2.82)	<0.001	37	3.31 (1.96-5.59)	<0.001
Positive												
INP 0	714	Reference		229	Reference		408	Reference		162	Reference	
INP 1	113	0.92 (0.67-1.25)	0.580	33	1.21 (0.74-2.00)	0.445	39	1.20 (0.74-1.93)	0.463	5	1.66 (0.51-5.37)	0.398
INP 2	411	1.27 (1.07-1.51)	0.006	73	1.16 (0.81-1.65)	0.417	347	1.46 (1.19-1.80)	<0.001	20	2.41 (1.29-4.48)	0.006
INP 3	440	1.65 (1.41-1.94)	<0.001	74	1.60 (1.15-2.22)	0.005	269	1.80 (1.45-2.24)	<0.001	19	2.86 (1.53-5.33)	0.001

Abbreviations: NLR, neutrophil-to-lymphocyte ratio; PLR, platelet-to-lymphocyte ratio; INP, integrated NLR and PLR; HR, hazard ratio; 95% CI, 95% confidence interval. Effect-size estimates were calculated under the COX proportional hazards regression models. $^{*}p$ was adjusted for age, gender, smoking, drinking, body mass index and family cancer history. Cutting value: 2.07 for NLR and 168.50 for PLR in overall patients; 2.36 for NLR and 159.23 for PLR in patients with esophageal cancer; 1.97 for NLR and 188.0 for PLR in patients with gastric cancer; 3.37 for NLR and 264.29 for PLR in patients with colorectal cancer. Definitions: INP 0: NLR ≤ cutting point and PLR ≤ cutting point; INP 1: NLR ≤ cutting point and PLR > cutting point; INP 2: NLR > cutting point and PLR ≤ cutting point; INP 3: NLR > cutting point and PLR > cutting point.

Supplementary Figure 1. The three-dimension surface displaying the combined association of NLR and PLR with the risk of digestive tract malignancy-specific morality in all study patients. Abbreviations: NLR, neutrophil-to-lymphocyte ratio; PLR, platelet-to-lymphocyte ratio.

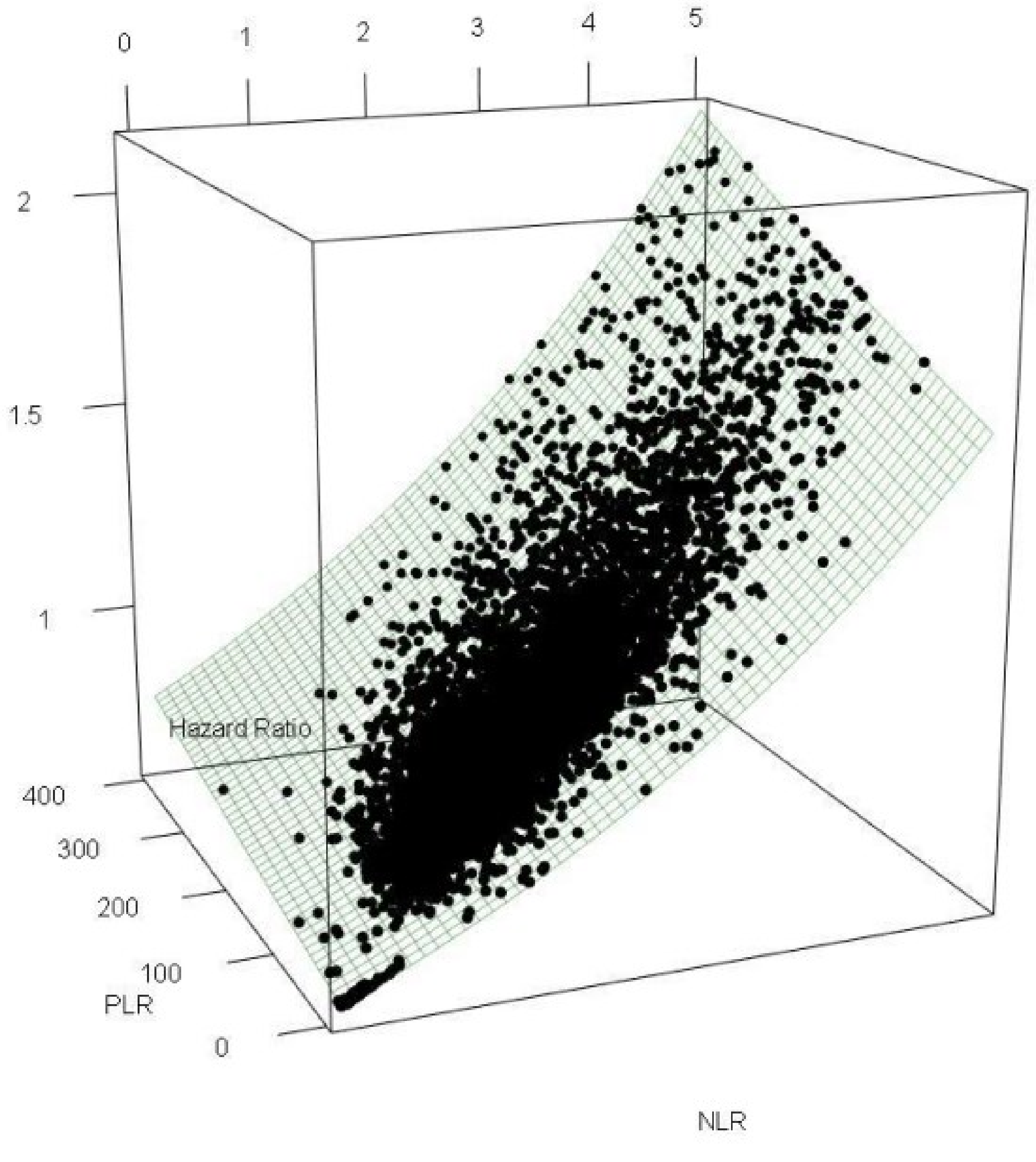

Supplement: Supplementary file 1 [file Data_Sheet_1.PDF]
